# Seforta, an integrated tool for detecting the signature of selection in coding sequences

**DOI:** 10.1186/1756-0500-7-240

**Published:** 2014-04-16

**Authors:** Salvatore Camiolo, Sara Melito, Giampiera Milia, Andrea Porceddu

**Affiliations:** 1 Dipartimento di Agraria, Università degli Studi di Sassari, Sassari 07100, Italy

**Keywords:** Codon bias, Translation optimization, Translational accuracy

## Abstract

**Background:**

The majority of amino acid residues are encoded by more than one codon, and a bias in the usage of such synonymous codons has been repeatedly demonstrated. One assumption is that this phenomenon has evolved to improve the efficiency of translation by reducing the time required for the recruitment of isoacceptors. The most abundant tRNA species are preferred at sites on the protein which are key for its functionality, a behavior which has been termed “translational accuracy”. Although observed in many species, as yet no public domain software has been made available for its quantification.

**Findings:**

We present here Seforta (Selection for Translational Accuracy), a program designed to quantify translational accuracy. It searches for synonymous codon usage bias in both conserved and non-conserved regions of coding sequences and computes a cumulative odds ratio and a Z-score. The specification of a set of preferred codons is desirable, but the program can also generate these. Finally, a randomization protocol calculates the probability that preferred codon combinations could have arisen by chance.

**Conclusions:**

Seforta is the first public domain program able to quantify translational accuracy. It comes with a simple graphical user interface and can be readily installed and adjusted to the user's requirements.

## Findings

In spite of the various mechanisms which have evolved to maintain mRNA translation accuracy, errors still arise at a rate of one every 10^3^-10^4^ codons [1]. Between ten and 50% of random residue substitutions compromise protein function through their effect on the product's three dimensional structure [2]. Coding sequence composition is expected to influence the rate of mistranslation errors [3,4]. Because multiple aminoacyl tRNAs compete with one another for loading at the ribosome acceptor sites, codons corresponding to the most abundant tRNAs (preferred codons) tend to be translated with the highest fidelity. In *E.coli*, it has been demonstrated that the frequency of amino acid residue errors at preferred codons is approximately ten fold lower than at other codons [5]. Missense errors are likely to induce their greatest deleterious effect when they occur in a region which is key for the protein's functionality. Thus preferred codons may be non-homogeneously distributed due to a variety of evolutionary constraints affecting different parts of the gene product. This phenomenon has been noted in a number of genomes and is referred to as “selection for translational accuracy” [6]. Despite this, there is as yet no public domain software available to quantify translational accuracy. Here we present such a program, which we have called Seforta (SElection FOR Translational Accuracy); it benefits from a simple graphical user interface, and is designed to uncover the signature of selection for accuracy together with the identification of an optimal codon set.

**Figure 1:**
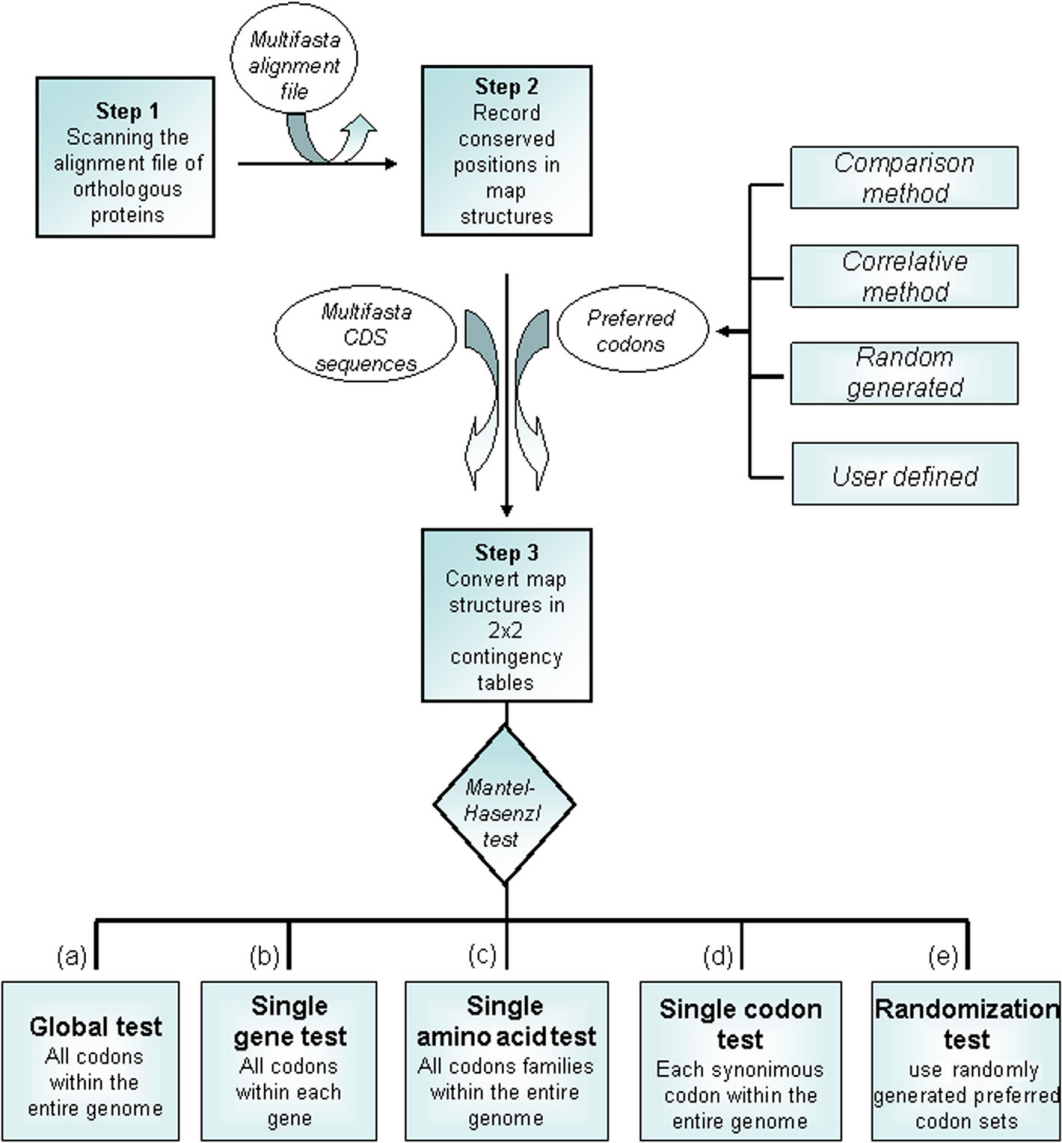
The Seforta workflow.

If natural selection biases codon usage to maximize translational accuracy, it is probable that preferred codons encode sites where a substitution would be deleterious [3]. Due to their importance for protein functionality, such sites are expected to be evolutionarily constrained and hence their position within coding sequences can be inferred by alignment between homologs [3]. Seforta analyzes whether the preferred codons are more frequent at these conserved sites by performing a test developed by H. Akashi [3]. The whole procedure is executed in three steps (Figure 1 and Additional file 1: Figure S1). First, each alignment of homologous proteins is scanned for conserved and non-conserved residue positions. Next, a set of independent 2x2 contingency tables summarizing the frequency of preferred codons at both the conserved and non-conserved residue positions is constructed (Additional file 1: Figure S1). Finally, a joint probability (for each residue in each gene analysed) is calculated following the Mantel Hasenzl procedure [7] (see Figure 1, option a). Alternatively a joint probability can be calculated for each gene individually (option b). It is also possible to compute the odds ratio for each residue across the entire genome (option c). Seforta allows the odds ratio to be calculated for each codon by considering it as the preferred one within a synonymous family (option d). The program also implements a routine which iteratively performs the Akashi test taking as input a randomly generated set of preferred codons (option e). In this way it becomes possible to identify the number and composition of the random sets of codons associated with a higher odd ratio than the chosen ones, and then allows this information to be used for significance testing [4].

Two alternative methods for the identification of preferred codons are available. The first is based on a comparison of codon frequencies between genes which are strongly or weakly expressed, using a conventional 2x2 contingency table analysis. Seforta identifies the two expression datasets to be compared by either selecting the two tails of the expression data distribution (based on a user-defined percentile value) or by dividing the dataset into a number of groups based on equal-sized intervals of expression level. When expression data are not available, the preferred codon lists can be identified via the correlation method proposed by Hershberg et al. [8,9]. This method identifies which codon(s) increase in frequency as genes become more biased in their codon usage. Seforta calculates the overall codon bias of a gene by using the effective number of codons (Nc’), which measures bias without any prior assumption regarding the identity of the preferred codons, while also controlling for background nucleotide composition [10]. The identification of preferred codons relies on the size of the Spearman correlation, having adjusted the overall level of codon bias for background nucleotide composition [8,9].

A test of Seforta on sequence data of *Drosophila melanogaster*[11] resulted in a perfect agreement with published statistics (Table 1). Of 6 million randomly chosen sets of preferred codons taken for the computation of the Akashi test, just four emerged with an association higher than the actual set of preferred codons, a result fully in line with the conclusions of Drummond and Wilke [11]. (Note that the latter authors performed the analysis using all possible codon sets, rather than using a randomization approach). Finally, we have used Seforta to calculate the signature of selection for translational accuracy for both each amino acid residue and each synonymous codon. The former analysis revealed different usage of preferred codons at selectively constrained sites for the analyzed amino acid residues (Additional file 2: Table S1). The single codon computation produced a positive and significant odds ratio for CGA, GGA, ACG and CGG, even though all these codons have been classified as non-preferred in *D. melanogaster*[11] (Additional file 2: Table S2). The synonymous codon test identified which residues in preferred codons are used most frequently at conserved sites, together with which codons appear to be under selection for translational accuracy. The analyses have suggested that fidelity and translation efficiency are not necessarily co-ordinated.

**Table 1:** **Comparison between Seforta output and a published test of *D. melanogaster *** **sequence data**[11]

**Method**	**Odds ratio**	**P (better codon set)**
Ref. [11]	1.362	2.72E-07
Seforta	1.362	6.60E-07

The size of the better codon set is reflected by a higher P value.

### Availability and requirements

**Project name:** seforta (version 0.1)

**Project home page:**http://sourceforge.net/projects/seforta/

**Operating system:** Linux 64-bit

**Programming language:** C++, Java

**Other requirements:** xterm must be installed

**License:** GNU GPL

### Availability of supporting data

The software source code together with test files are available at the Project home page reported above.

## Competing interests

The authors declare that they have no competing interests.

## Authors’ contributions

SC was involved in the design and realization of the software. SM and GM contributed to its testing and to correcting the algorithm. AP contributed to the conception of the project and participated in the drafting of the manuscript. All authors read and approved the final manuscript.

## Supplementary Material

Additional file 1: Figure S1 The Seforta procedure. In step 1 the sequence alignment file is scanned and the sequences of conserved residues (C), variable sites (V) and gapped sites (G) are used to construct a 2x2 contingency table based on the usage of preferred/non-preferred codons (step 2). The 2x2 table is an example of a Leucine contingency table relative to the gene fragment highlighted in the box. Click here for file

Additional file 2: Table S1 Amino acid under selection for accuracy in *D. melanogaster*. **Table S2:** Single codon test performed by Seforta on *D. melanogaster*. Codons that proved to be significantly over used in conserved sites are represented in bold. Codons that proved to be involved in the selection for translational accuracy while being “not preferred” according to previous studies are underlined. Click here for file
