# Supplementary figures and images for: Seforta, an integrated tool for detecting the signature of selection in coding sequences
Source: BMC Res Notes. 2014 Apr 16;7:240. doi: 10.1186/1756-0500-7-240 (PMC4022393; doi:10.1186/1756-0500-7-240)

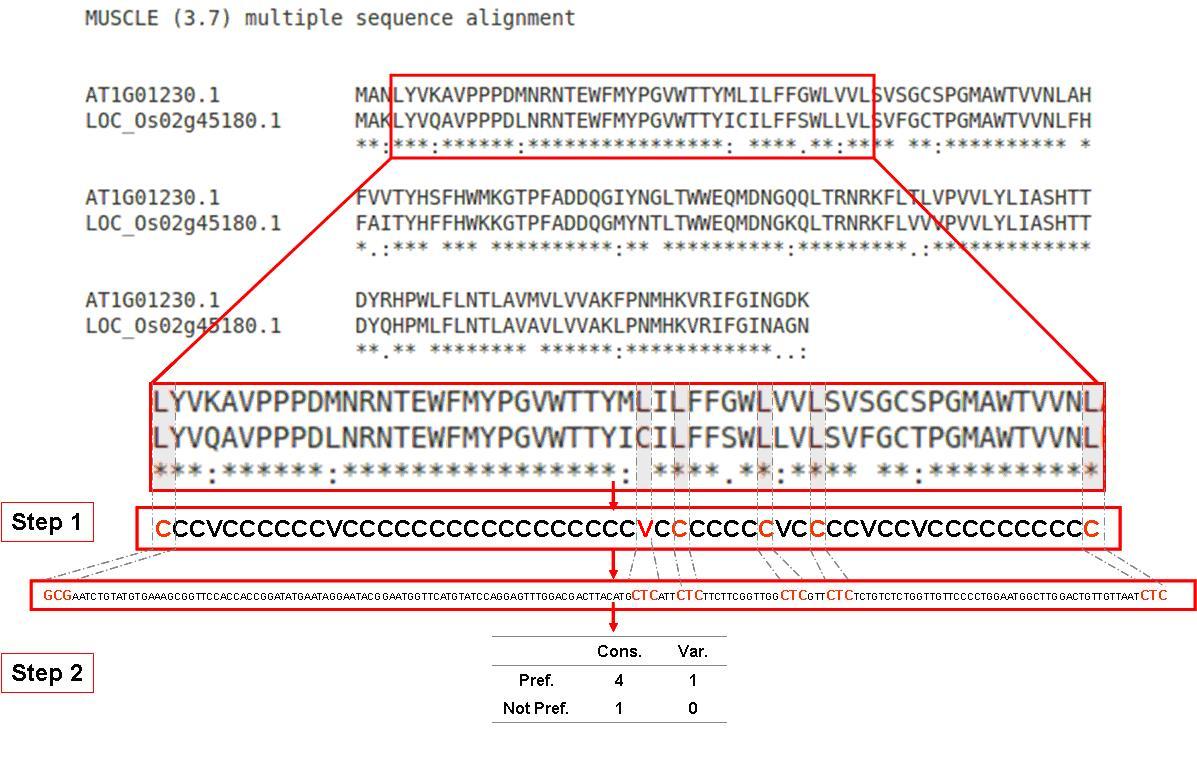

Supplement: Additional file 1: Figure S1 — The Seforta procedure. In step 1 the sequence alignment file is scanned and the sequences of conserved residues (C), variable sites (V) and gapped sites (G) are used to construct a 2x2 contingency table based on the usage of preferred/non-preferred codons (step 2). The 2x2 table is an example of a Leucine contingency table relative to the gene fragment highlighted in the box. [file 1756-0500-7-240-S1.jpeg]
