# Supplementary material for: Seforta, an integrated tool for detecting the signature of selection in coding sequences
Source: BMC Res Notes. 2014 Apr 16;7:240. doi: 10.1186/1756-0500-7-240 (PMC4022393; doi:10.1186/1756-0500-7-240)
Supplement: Additional file 2: Table S1 — Amino acid under selection for accuracy in D. melanogaster. Table S2: Single codon test performed by Seforta on D. melanogaster. Codons that proved to be significantly over used in conserved sites are represented in bold. Codons that proved to be involved in the selection for translational accuracy while being “not preferred” according to previous studies are underlined. [file 1756-0500-7-240-S2.doc]

**Supplemental material**

**Table S1.** Amino acid under selection for accuracy in *D. melanogaster*

| **Amino acid** | **Odd Ratio** |
| --- | --- |
| R | 2.41* |
| L | 1.87* |
| E | 1.68* |
| A | 1.47* |
| V | 1.51* |
| Q | 1.55* |
| S | 1.31* |
| K | 1.47* |
| I | 1.32* |
| H | 1.38* |
| P | 1.22* |
| Y | 1.30* |
| C | 1.40* |
| F | 1.15* |
| D | 1.10* |
| N | 1.07* |
| G | 1.02 |
| T | 1.01 |

*p<0.05

**Table S2:** Single codon test performed by Seforta on *D. melanogaster*. Codons that proved to be significantly over used in conserved sites are represented in bold. Codons that proved to be involved in the selection for translational accuracy while being “not preferred” according to previous studies are underlined.

| **Amino acid** | **Codon** | **Odd ratio** | **Z-score** |  | **Amino acid** | **Codon** | **Odd ratio** | **Z-score** |
| --- | --- | --- | --- | --- | --- | --- | --- | --- |
| A | GCA | 0.751571 | 15.78*** |  | N | AAT | 0.930994 | 3.78* |
| A | **GCC** | 1.46706 | 24.49*** |  | P | CCA | 0.906782 | 4.21** |
| A | GCG | 0.753178 | 15.78*** |  | P | **CCC** | 1.21526 | 8.27** |
| A | GCT | 1.01769 | 0.92 |  | P | CCG | 1.04788 | 1.97 |
| C | **TGC** | 1.39657 | 7.34** |  | P | CCT | 0.798638 | 7.98** |
| C | TGT | 0.716038 | 7.34** |  | Q | CAA | 0.645809 | 21.02*** |
| D | **GAC** | 1.09534 | 4.76** |  | Q | **CAG** | 1.54845 | 21.02*** |
| D | GAT | 0.912962 | 4.76** |  | R | AGA | 0.459329 | 28.51*** |
| E | GAA | 0.596013 | 29.15*** |  | R | AGG | 0.360308 | 41.46*** |
| E | **GAG** | 1.67782 | 29.16*** |  | R | **CGA** | 1.55297 | 13.15*** |
| F | **TTC** | 1.15362 | 4.99** |  | R | **CGC** | 2.37466 | 29.29*** |
| F | TTT | 0.86684 | 4.98** |  | R | **CGG** | 1.18515 | 5.24** |
| G | **GGA** | 1.43519 | 14.92*** |  | R | **CGT** | 1.59769 | 13.11*** |
| G | GGC | 1.01596 | 0.73 |  | S | AGC | 0.922269 | 5.18** |
| G | GGG | 0.61864 | 15.37*** |  | S | AGT | 0.91413 | 4.85** |
| G | GGT | 0.854676 | 6.42** |  | S | TCA | 0.794091 | 10.89*** |
| H | **CAC** | 1.37751 | 11.90*** |  | S | **TCC** | 1.23514 | 12.70*** |
| H | CAT | 0.725945 | 11.90*** |  | S | **TCG** | 1.21331 | 10.59** |
| I | ATA | 0.685601 | 17.60*** |  | S | TCT | 0.838765 | 7.83** |
| I | **ATC** | 1.32049 | 14.28*** |  | T | ACA | 0.985077 | 0.74 |
| I | ATT | 1.01276 | 0.64 |  | T | ACC | 1.01105 | 0.65 |
| K | AAA | 0.679969 | 19.00*** |  | T | **ACG** | 1.13658 | 6.74** |
| K | **AAG** | 1.47066 | 19.00*** |  | T | ACT | 0.861661 | 7.49** |
| L | CTA | 1.02319 | 0.79 |  | V | GTA | 0.644484 | 18.47*** |
| L | CTC | 1.01941 | 0.76 |  | V | GTC | 0.736799 | 15.72*** |
| L | **CTG** | 2.01472 | 35.43*** |  | V | **GTG** | 1.86557 | 34.72*** |
| L | CTT | 0.797935 | 8.77** |  | V | GTT | 0.792173 | 11.38*** |
| L | TTA | 0.426656 | 30.34*** |  | Y | **TAC** | 1.29884 | 7.62** |
| L | TTG | 0.695216 | 18.25*** |  | Y | TAT | 0.769915 | 7.62** |
| N | **AAC** | 1.07412 | 3.78* |  |  |  |  |  |

*p<0.01, **p<0.05, ***p<0.001.
